# Validation of the Grief Support in Healthcare Scale among frontline nursing professionals working in COVID-19 inpatient wards in Korea

**DOI:** 10.3389/fpsyt.2023.1097022

**Published:** 2023-04-20

**Authors:** Junseok Ahn, Young Rong Bang, Eulah Cho, Oli Ahmed, Jeong Hye Kim, Youjin Hong, Seockhoon Chung, Keith A. Anderson

**Affiliations:** ^1^Department of Psychiatry, Ulsan University Hospital, University of Ulsan College of Medicine, Ulsan, Republic of Korea; ^2^Department of Psychiatry, Asan Medical Center, University of Ulsan College of Medicine, Seoul, Republic of Korea; ^3^Department of Psychology, University of Chittagong, Chattogram, Bangladesh; ^4^National Centre for Epidemiology and Population Health, Australian National University, Canberra, ACT, Australia; ^5^Department of Clinical Nursing, University of Ulsan, Seoul, Republic of Korea; ^6^Department of Psychiatry, GangNeung Asan Hospital, University of Ulsan College of Medicine, Gangneung, Republic of Korea; ^7^Department of Social Work, University of Mississippi, Oxford, MI, United States

**Keywords:** COVID-19, grief, nurses, depression, anxiety

## Abstract

**Introduction:**

During the COVID-19 pandemic, healthcare workers (HCWs) have been exposed to higher levels of anxiety and psychological stress than the general population. Nurses who cared for COVID patients could not avoid repeated mourning as they witnessed the deaths of their patients. Therefore, tools are needed to evaluate whether there is adequate support for the grieving process of HCWs in both qualitative and quantitative manners.

**Methods:**

Data from 229 nurses who witnessed the deaths of COVID-19 inpatients were analyzed using an online survey of nurses working in three tertiary hospitals. Factor analysis was conducted to validate the 10-item Korean version of Grief Support in Healthcare Scale (GSHCS). Stress and Anxiety to Viral Epidemics-9 was used to measure stress and anxiety caused by coronavirus, Generalized Anxiety Disorder-7 was used to measure overall anxiety, and Patient Health Questionnaire-9 was used for depression. Convergent validity correlation analysis was also performed with GSHCS.

**Results:**

The two-factor model showed a good fit for the 10-item GSHCS (*χ*^2^ = 35.233, df = 34, *p* = 0.410, CFI = 0.999, TLI = 0.990, RMSEA = 0.013, SRMR = 0.064). Cronbach’s alpha is 0.918 and McDonald’s omega is 0.913, suggesting that the 10-item version of the GSHCS is reliable for determining psychometric properties.

**Conclusion:**

According to this study, the 10-item Korean version of the GSHCS is a reliable and valid measure of psychological support for grief among frontline nursing professionals who have witnessed the deaths of patients they cared for while working in COVID-19 inpatient wards. A two-factor model of the GSHCS has a good model fit and good convergent validity with other rating scales that measure viral anxiety, depression, and general anxiety.

## Introduction

With the first COVID-19 case reported in December 2019 (1), the virus has spread rapidly worldwide. Approximately 531 million cases and 6.3 million deaths have been reported worldwide, with 18 million confirmed and 24,323 deaths reported in South Korea by June 2022 (2). There is a high prevalence of psychological issues such as depression, anxiety, insomnia, and post-traumatic stress disorder in the general population (3). It is important to note that the South Korean government recently announced a policy of coping with Coronavirus using a “Step by Step Recovery” strategy; however, this policy has been halted since a rise in the number of confirmed cases of the Omicron variant has occurred (4).

### Psychological distress of healthcare workers in the COVID-19 pandemic

As healthcare workers struggled at the frontline of COVID-19, they were more vulnerable to psychological distress than the general population. They suffered from anxiety that they could become infected themselves as well as fear that they could spread the infection to family and friends. During the pandemic era, they also complained of work-related stress due to increased work intensity resulting from an explosion in the workload due to the rapid increase in the number of infected populations (5). However, the big difference between healthcare workers (HCWs) and the general population is that HCWs are forced to experience repetitive grief. In general, HCWs are more likely to see death than the general population, but medical staff working on the frontlines of COVID-19 during the pandemic had to experience a higher rate of patient death than their usual work experiences. During this period, death due to the rapid worsening of symptoms was common in elderly patients or patients with existing chronic diseases, and many HCWs complained of helplessness and guilt for not being able to save the patient.

### Grief reaction of and grief support for nursing professionals

Nurses frequently experience the deaths of their patients and are highly exposed to grief. Nurses report grief emotions, such as sadness, fear, guilt, and powerlessness (6). Leaving the emotional experiences of nurses can lead to negative consequences on the physical, emotional, and spiritual health of patients when death and suffering occur repeatedly. However, nurses do not have the time to deal with losses, and they lack training in how to process their emotions. On the other hand, most nurses attempt to overcome this problem (7). There is a lack of policy support and education regarding grief management that can help nurses manage grief and loss after the death of their patients. Therefore, nurses need support to overcome the grief of recurrent patient deaths and maintain professionalism (6).

During the COVID-19 pandemic, family visits to medical institutions were restricted due to patient safety, and nurses were the only point of contact or support for many patients who died without seeing family or friends (8, 9). Traditionally, Korean funerals last 3 to 5 days. Family members spent 2 nights and 3 days together at the funeral home. However, due to the rapid increase in deaths and social distancing caused by the pandemic, most funerals took place in less than 3 days, and only a few people attended. There was no specific prohibition in the United States against being in a room with a dead body, but it is forbidden to be in the same room as an infected person in Korea. Moreover, burials and cremations were not restricted in the United States, but burials were prohibited in Korea. The Korean guidelines of prohibitions regarding funerals were maintained until relatively recently, which indicates a difference in time period. The guideline was amended in January 2022, but approximately 6,000 bereaved families had to say goodbye to their loved ones without saying goodbye.

Instead family members, nurses provide post-mortem care such as closing the eyes of the dead patient, wiping the body, and changing the shroud to respect the patient’s dignity. However, during the COVID-19 pandemic, such nursing care is limited owing to concerns about infection, which added to the emotional distress and grief of nurses (6).

Support from others during the grieving process may have a significant influence on nurses’ grief (10) and facilitate healing. In patient–nurse relationships, nurses must maintain their professional distance. As a result, their grief may go unrecognized and needed support may be unavailable to them. In end-of-life care, nurses practice emotional distancing to limit their experience of grief and protect the nursing profession. On the other hand, professional identity and responsibility as nurses often prevent emotional support for patients (11). Social support, including the psychological support of colleagues who are best known about their situation relating to the death of their patients, is very important for nurses to overcome grief due to the death of a patient (12). During the COVID-19 pandemic, the support of colleagues and family members decreased owing to the increased demand from patients and the burden of nursing work (9). Additionally, nurses recognized that unexpected death was the most difficult problem. When caring for a dying patient, a nurse should feel supported, including through interactions with colleagues, and be given the opportunity to express their feelings and experiences. In addition, organizational support is needed for nurses’ psychological and personal well-being (13). Since many infected patients died unexpectedly in the COVID-19 wards during this pandemic, developing grief support tools for frontline HCWs is justified.

During the COVID-19 pandemic, it is imperative that HCWs receive psychological or environmental support for their grief. The Grief Support in Healthcare Scale (GSHCS) is a rating scale which can assess support for healthcare workers’ grief reaction, and was developed using the following components of the theory of disenfranchised grief focused on support: recognition of the relationship, acknowledgment of the loss, and inclusion of the griever (14). Lee et al., developed and validated a pandemic grief scale during the pandemic period, which has been validated in several countries, and they also standardized pandemic grief scale it for HCWs (15). The GSHCS can provide a more comprehensive measure of grief support by incorporating the diversity of sources and forms available to frontline nursing professionals. The purpose of this study was to examine the reliability and validity of the GSHCS among nursing professionals working in COVID-19 inpatient wards who encounter death in the patients they care for.

## Methods

### Participants and procedures

An online survey was conducted at three tertiary-level affiliated hospitals of the University of Ulsan, including Asan Medical Center in Seoul, Ulsan University Hospital in Ulsan, and GangNeung Asan Hospital in Gangneung, from April 7 to 26, 2022. The online survey was distributed to frontline nursing professionals working in COVID-19 inpatient wards in the three hospitals. We obtained the participants’ age, sex, and marital status, but no identifiable personal information was collected. In addition, we gathered responses to questions on COVID-19, such as “Did you witness any deaths of patients caused by COVID-19 while working on COVID-19 inpatient wards?” “Did you experience being quarantined due to COVID-19 infection?,” “Did you experience being infected with COVID-19?” or “Did you get vaccinated?.” Psychiatric history and current psychiatric distress were assessed.

We developed an e-survey form according to the Checklist for Reporting Results of Internet e-Surveys (CHERRIES) guidelines (16), and the investigators checked the usability and technical functionality before implementing the survey. All 339 (239 in Asan Medical Center, 150 in Ulsan University Hospital, and 50 in GangNeung Asan Hospital) nursing professionals were working in COVID-19 inpatient wards in each hospital, and we targeted to collect responses from at least 60% (*N* = 203) of the eligible population. We collected 266 (143 in Asan Medical Center, 94 in Ulsan University Hospital, and 29 in GangNeung Asan Hospital) responses. Out of the 266 respondents, 229 reported that they witnessed the death of patients (126, 85, and 18) who they took care of, which were finally enrolled for statistical analysis.

COVID-19 reached its fifth peak in Korea in March 2022, and our survey was conducted in the duration of fifth waves nationally and globally while epidemic was mainly due to Omicron variant (2, 17).

The study protocol was approved by the Institutional Review Boards (IRBs) of the Asan Medical Center (2022–0323), Ulsan University Hospital (UUH 2022–02–016-003), and GangNeung Asan Hospital (2022–03–003-001), which waived the requirement for written informed consent.

## Measures

### Grief Support in Healthcare Scale

The Grief Support in Healthcare Scale (GSHCS) was designed to evaluate the support available to HCWs in the event of grief. It was developed based on the theory of disenfranchised grief that emphasizes support (18). It has 15 items and three subscales: Factor I, recognition of the relationship; Factor II, acknowledgment of the loss; and Factor III, inclusion of the griever. Each item was rated on a 5-point Likert scale, with responses ranging from 1 (strongly disagree) to 5 (strongly agree).

We translated the original English version of the GSHCS into Korean using translation and back-translation methods. The original English version of the GSHCS (Supplementary File 1) was translated into the Korean version (Supplementary File 2) by a bilingual expert. An additional bilingual expert translated the Korean text back into the original English text without referring to the original English text. The reverse-translated English version was compared and verified by a third party, and subtle differences were observed. By completing these steps, we created the Korean version of the GSCHS.

In this study, we explored a 10-item version of the GSHCS. We excluded items 11–15, which are not applicable in terms of the healthcare system or social norms in South Korea. HCWs do not usually attend their patient’s funeral in Korea.

### Stress and Anxiety to Viral Epidemics-9

The Stress and Anxiety to Viral Epidemics-9 (SAVE-9) scale can measure distress and anxiety experienced by healthcare workers during the COVID-19 pandemic (19). Items in the SAVE-9 were originally grouped into two categories: Factor I (items 1–5 and 8), anxiety about the epidemic, and Factor II (items 6–7 and 9), work-related stress associated with the epidemic. Each item on the SAVE-9 is rated on a 5-point Likert scale ranging from 0 (never) to 4 (always), with higher scores indicating greater levels of stress and anxiety. The Korean version of the SAVE-9, originally developed in Korea, was used in this study. Cronbach’s alpha was 0.826 for this sample.

### Generalized Anxiety Disorder-7

The Generalized Anxiety Disorder-7 (GAD-7) is a self-report scale that measures general symptoms (20). The scale contains seven items rated from zero (never at all) to three (almost every day). A high total score indicates a high degree of anxiety. In this study, we used the Korean version of the GAD-7 (21), and Cronbach’s alpha was 0.934.

### Patient Health Questionnaire-9

The Patient Health Questionnaire-9 (PHQ-9) is a self-rating scale used to measure depression severity (22). The survey has nine items that can be rated from 0 (never) to 3 (nearly every day). A higher total score indicated more severe depression. In this study, we used the Korean version of the PHQ-9 (23), and Cronbach’s alpha was 0.880.

## Statistical analysis

The distribution of responses was evaluated using descriptive statistics (percentages, means, and standard deviations).

Second, confirmatory factor analysis (CFA) with a diagonal weighted least square estimation method was conducted to confirm the construct validity of the 10-item GSHCS. Before conducting EFA, the normality assumption was checked based on skewness and kurtosis (within the range ± 2 (22)). The Kaiser–Meyer–Olkin (KMO) value and Bartlett’s test of sphericity were examined to explore data suitability and sampling adequacy for factor analysis. A satisfactory model fit was based on the values of a standardized root-mean-square residual (SRMR) ≤ 0.05, a root-mean-square error of approximation (RMSEA) ≤ 0.10, and comparative fit index (CFI) and Tucker–Lewis index (TLI) ≥ 0.90 (24, 25). Multi-group CFAs with configural, metric, or scale-invariant models were conducted to examine whether the 10-item GSHCS could assess the degree of support in the same way among frontline nursing professionals with viral anxiety (SAVE-9 ≥ 22), depression (PHQ-9 ≥ 10), or anxiety (GAD-7 ≥ 10).

Second, reliability was assessed using Cronbach’s alpha, McDonald’s Omega, and split-half reliability (odd-even). Pearson’s correlation was performed to examine the convergent validity of the SAVE-9, PHQ-9, and GAD-7 scales. The psychometric properties were assessed using the Rasch model. In the Rasch model, the infit mean square (MnSQ), outfit MnSQ, item difficulty, item and person separation index, and item and person reliability were estimated. SPSS version 21.0, AMOS version 27 (SPSS, Inc., Chicago, Illinois, United States), RStudio, and jMetrik software (version 4.1.1) were used for statistical analysis.

## Results

All 229 responses from nursing professionals working in COVID-19 inpatient wards were analyzed. The majority of the respondents were female (n = 216) and had worked at each hospital for an average of 6.9 years. Most of the respondents (95%) were shift workers. Among the participants, 41.5% were quarantined due to COVID-19 and 37.1% were infected with COVID-19. Table 1 displays other demographics and rating scale scores.

**Table 1:** Clinical characteristics of participants (*N* = 229).

Variables	*N* (%), Mean ± SD
*Sex (female)*	216 (94.3%)
*Age*	30.1 ± 6.3
*Years of employment*	6.9 ± 6.0
Marital Status*
Single	175 (76.4%)
Married, without kids	17 (7.4%)
Married, with kids	35 (15.3%)
*Are you a shift worker?*	218 (95.2%)
*Questions on COVID-19*
Are you taking care of COVID-19 infected patients? (Yes)	229 (100.0%)
Did you experience being quarantined due to infection with COVID-19? (Yes)	95 (41.5%)
Did you experience being infected with COVID-19? (Yes)	85 (37.1%)
Did you get vaccinated? (Yes)	229 (100.0%)
Did you experience deaths of COVID-19 infected patients? (Yes)	229 (100.0%)
*Psychiatric history*
Did you have experience or treated depression, anxiety, or insomnia? (Yes)	37 (16.2%)
Now, do you think you are depressed or anxious, or do you need help for your mood state? (Yes)	33 (14.4%)
*Rating scales scores*
10-item Grief Support in Healthcare Scale	28.9 ± 7.5
Subscale 1–Recognition of the Relationship	14.7 ± 4.2
Subscale 2–Acknowledgment of the Loss	14.2 ± 4.2
Stress and Anxiety to Viral Epidemics-9 items	21.5 ± 6.3
Generalized Anxiety Disorders-7 items	4.2 ± 4.8
Patient Health Questionnaire-9 items	8.1 ± 5.3

*Two values were missing.

### Confirmatory factor analysis

The item-level properties of the Korean GSHCS are listed in Table 2. Sampling was adequate, and data were suitable for factor analysis (KMO value of 0.81 and Bartlett’s test of sphericity value *p* < 0.001, Table 3). Corrected item-total correlations ranged from.633 to.851, which was above the minimum cutoff value (0.30).

**Table 2:** Item properties of the 10-item Korean version of GSHCS.

Items	Descriptive				CITC	CID	Factor loading
	*M*	SD	Skewness	Kurtosis			
Item 1	2.841	1.005	−0.218	−0.460	0.757	0.882	0.818
Item 2	2.875	0.988	−0.209	−0.328	0.834	0.865	0.870
Item 3	3.237	0.998	−0.359	−0.071	0.775	0.878	0.788
Item 4	3.082	1.005	−0.449	−0.082	0.795	0.874	0.822
Item 5	2.690	0.971	−0.174	−0.603	0.633	0.907	0.727
Item 6	2.905	0.971	−0.266	−0.069	0.778	0.895	0.797
Item 7	2.905	0.967	−0.272	−0.024	0.851	0.880	0.885
Item 8	3.009	0.998	−0.281	−0.063	0.807	0.889	0.862
Item 9	2.828	0.974	−0.328	−0.220	0.823	0.886	0.876
Item 10	2.569	0.933	−0.203	−0.389	0.647	0.921	0.704

M, mean; SD, standard deviation; CITC, corrected item-total correlation; CID, Cronbach’s alpha if item deleted; CFA, Confirmatory factor analysis.

**Table 3:** Scale-level psychometric properties of the 10-item Korean version of GSHCS.

Psychometric properties	Scores			Suggested cut off
	Subscale 1	Subscale 2	Full scale	
Cronbach’s alpha	0.903	0.914	0.918	≥ 0.7
McDonald’s Omega	0.904	0.915	0.913	≥ 0.7
Split-half reliability (odd-even)	0.956	0.944	0.969	≥ 0.7
Composite reliability	0.903	0.915		≥ 0.7
Standard error of measurement	1.313	1.225		< SD /2
Item separation index	4.387	3.552		≥ 2
Person separation index	2.785	2.528		≥ 2
Item reliability	0.951	0.927		≥ 0.7
Person reliability	0.886	0.865		≥ 0.7
KMO measure of sample adequacy	0.81			0.50
Bartlett’s test of sphericity	023.381 (<0.001)			Significant
*Model fits of confirmatory factor analysis*				
*χ*^2^ (df, *p* value)	35.233 (34, 0.410)			Nonsignificant
CFI	0.999			>0.95
TLI	0.990			>0.95
RMSEA	0.013			<0.08
SRMR	0.064			<0.08

**Figure 1:**
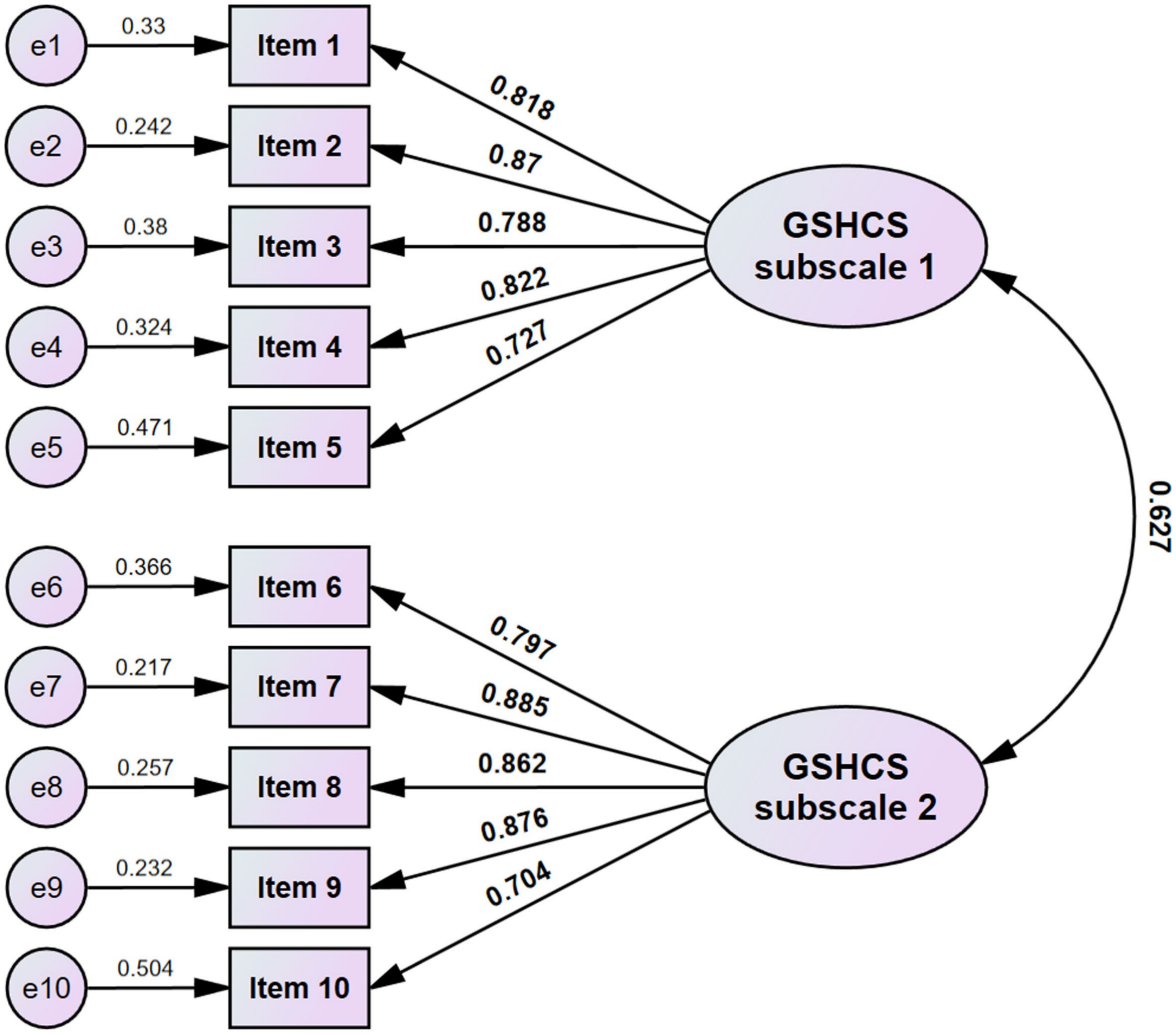
Factor structure of 10-item Korean version of the GSHCS.

CFA revealed a good fit for the two-factor model of the 10-item version of the GSHCS (*χ*^2^ = 35.233, df = 34, *p* value = 0.410, CFI = 0.999, TLI = 0.990, RMSEA = 0.013, SRMR = 0.064) (Table 3). Factor loadings in the CFA ranged between 0.727 and 0.870 for subscale 1 and 0.704 and 0.885 for subscale 2 (Figure 1). Based on the results of the multi-group CFA with a configural, metric, or scale-invariant model, we observed that the 10-item version of the GSHCS could measure the degree of support in the same way across viral anxiety, depression, or generalized anxiety (Supplementary Table 1).

### Reliability of the 10-item version of the GSHCS and evidence-based relationships with other variables

A full-scale Cronbach’s alpha of 0.918 and McDonald’s omega of 0.913 indicate that the 10-item version of the GSHCS is reliable (Table 3). Subscales 1 (Cronbach’s alpha = 0.903, McDonald’s omega = 0.904) and 2 (Cronbach’s alpha = 0.914, McDonald’s omega = 0.915) also showed good internal consistency. The 10-item version of GSHCS showed a good convergent validity with SAVE-9 (*r* = 0.150, 95% CI [0.021, 0.275], *p* = 0.023), PHQ-9 (*r* = 0.145, 95% CI [0.016, 0.269], *p* = 0.028), and GAD-7 (*r* = 0.155, 95% CI [0.026, 0.279], *p* = 0.019). Among the subscales, subscale 1 (recognition of the relationship) was not significantly correlated with SAVE-9 (*r* = 0.082, *p* = 0.216), PHQ-9 (*r* = 0.086, *p* = 0.196), and GAD-7 (*r* = 0.064, *p* = 0.334); however, subscale 2 (acknowledgment of the loss) was significantly correlated with SAVE-9 (*r* = 0.166, *p* = 0.012), PHQ-9 (*r* = 0.146, *p* = 0.027), and GAD-7 (*r* = 0.132, *p* = 0.047).

### Rasch model

Supplementary Table 2 present the Rasch model outputs for the GSHCS scale. The fit and outfit mean squares of all the items are between the recommended ranges (0.50–1.50) except for item 10. The outfit mean square of item 10 was 1.55, which is above the recommended range, but the outfit mean square was 1.49, which is between the recommended ranges; therefore, this factor should be considered. Regarding item difficulty, item 3 had the lowest item difficulty and item 5 had the highest item difficulty in the recognition of the relationship subscale. In the “acknowledgement of the loss” subscale, item 8 has the lowest item difficulty and item 10 has the highest item difficulty. Item and person reliability and separation indices were above the recommended cutoff (≥ 0.80, reliability, and ≥ 2 for separation indices).

## Discussion

In this study, we found that the 10-item Korean version of the GSHCS is a reliable and valid rating scale for measuring psychological support for the grief of frontline nursing professionals who witnessed the deaths of patients they care for while working in COVID-19 inpatient wards. We found a good model fit for a two-factor model of the GSHCS and good convergent validity with other rating scales that can measure viral anxiety, depression, or general anxiety. However, we found that subscale 2 (acknowledgment of the loss) was significantly associated with the level of psychological distress among frontline nursing professionals, whereas subscale 1 (recognition of the relationship) was not.

In this study, we also found good model fits for a two-factor model of the GSHCS, including subscales 1 (recognition of the relationship) and 2 (acknowledgment of the loss). In Doka’s study, one of the factors supporting disenfranchised grief was the well-understood relationship between the HCW and the patient they cared for. Recognizing a relationship (subscale 1) was not associated with depression, viral anxiety, or general anxiety. Interestingly, this pattern is similar to that of the original study, which did not find any significant correlation between distress from grief and depression. However, acknowledgment of loss (subscale 2) was highly correlated with depression, viral anxiety, and general anxiety. As in the previous study, it was confirmed again in the present study that it is important to know that the patients have died in order to reduce grief support for disenfranchised grief among HCWs. In general, patient information is not freely shared by HCWs with others because of confidentiality issues. Furthermore, medical personnel on the frontline of COVID-19 often had an atmosphere of hiding instead of freely expressing their concerns about the disease. In other words, it is both difficult to say that HCWs in the COVID-19 period had an intimate relationship with patients and that their work environment permitted them to discuss their relationship adequately. We found that the psychological distress that HCWs suffered from disenfranchised grief which was associated with COVID-19 depends primarily on their awareness of the loss they are experiencing rather than whether others are aware of their intimate relationship with the patient.

We excluded subscale 3 (including the griever) from the final model in this study. In addition, hospital death has not been considered a good death, which is called “Gaeksa; death outside of the house.” Koreans have traditionally viewed dying at home with family watching as a good death (26). Grief support includes more than social support. This can also include letting individuals participate in rituals or allowing them to mourn privately. Because medical professionals are people who live in cultures before they become medical professionals, there are differences in how they view the loss of patients in cultures that view funerals differently. As a natural process of life, death is not easy to talk about in Korea and is considered unacceptable by patients and their families as well. It is said that American Koreans and Hispanics are forbidden from discussing death with patients because they could negatively affect them (27).

We also examined the convergent validity of the GSHCS for viral anxiety during the pandemic. The death of a COVID-19 patient always occurs in a hospital that requires isolation. Until December 2021, if infected patients died during COVID-19, burials were forbidden, and funerals were mandatory within 24 h after cremation in Korea. Family members could not have direct contact with the deceased and were just allowed to watch their bodies enter the crematorium in an isolated or remote location. Consequently, HCWs or professional funeral directors handled most of the funeral procedures originally handled by the bereaved family. When a patient dies during the COVID-19 pandemic, the medical staff has no choice but to be semi-compulsively exposed to the death and mourning processes of more patients than usual. In addition, a medical professional who shares the same culture would suffer great psychological stress if it led to an unavoidable situation that might lead to disenfranchised grief in bereaved families.

Grieving was often incomplete in this COVID-19 pandemic (28), and mourning was insufficient even before the COVID-19 pandemic because the death of patients was regarded as a failure by HCWs (29). The excessive workload of HCWs often results in insufficient mourning of long-term patients. A mourning reaction combined with the excessive work of these medical staff members can cause severe burnout or even major psychiatric disorders. During the severe acute respiratory syndrome (SARS) era, Rober et al. reported that HCWs in Toronto were significantly vulnerable to burnout or post-traumatic stress disorder 13–26 months after the SARS breakout (30). During the COVID-19 pandemic, critically ill patients faced high mortality rates, and HCWs did not have time to grieve about the loss of their individual patients. Due to the longer duration of the pandemic, many of these imperfect mourning events might have continuously accumulated. Moreover, medical staff are often forcibly assigned to related tasks in COVID-19 situations, allowing them to be exposed to unintentional losses in emergency situations. Lastly, it was difficult for medical staff to share their condolences with their friends when caring for COVID-19 patients. The medical staff in charge of COVID-19 were often isolated from their interpersonal relationships because of the risk of spreading the disease to others. For this reason, they were also in a difficult position to inform family and friends about work-related facts and to comfort them about carrying out COVID-19-related tasks. Furthermore, the medical staff felt the survivor’s guilt when their patients died, but they survived (28). During this rapid pandemic, medical staff have continued to work in an environment where there is an inadequate psychological support system for the death of a patient.

This study has several limitations. First, this study was conducted among HCWs working in COVID-19 inpatient units. As all participants experienced the death of infected patients, the results of this study may not be generalizable to other situations where HCWs can experience the death of patients regardless of whether they are infected with COVID-19. Second, this study was conducted as an anonymous online survey rather than face-to-face interview, to prevent viral transmissions. Psychological stress may be underreported because self-report responses are subject to bias. Third, the survey was conducted in three tertiary-level hospitals in Korea; however, there might be differences in the hospital environment. Furthermore, the sample size was not large, even though we completed the survey in three hospitals. However, this sample was homogeneous, since all participants were working in COVID-19 inpatient units.

In conclusion, we confirmed that the 10-item GSHCS is a reliable and valid measure of psychological support for frontline nursing professionals’ grief. Psychological support is needed for nursing professionals who experience death while working in COVID-19 inpatient units, and it is important to assess whether they can provide psychological support for the grief that they experience. Equally important is the development and application of resources to support healthy grieving among nurses and other healthcare professionals. Fortunately, there are existing programs to support grief in these settings, including resources that were developed specifically to address grief during the COVID-19 pandemic (27). It is also critically important that we augment the training on grief and grief support for students in the healthcare professions. In training and supporting both current and future healthcare professionals, we may see improved scores on the GSHCS and higher levels of emotional well-being in our healthcare workforce.

## Data availability statement

The raw data supporting the conclusions of this article will be made available by the authors, without undue reservation.

## Ethics statement

The study protocol was approved by the Institutional Review Boards (IRBs) of the Asan Medical Center (2022–0323), Ulsan University Hospital (UUH 2022–02–016-003), and GangNeung Asan Hospital (2022–03–003-001), which waived the requirement for written informed consent. The patients/participants provided their written informed consent to participate in this study.

## Author contributions

SC, YH, and JA: conceptualization, investigations, resources, and project administration. SC, JA, YB, EC, and YH: data curation. SC, OA, YB, and JA: formal analysis. YB, JK, and OA: methodology. EC: visualization. JA, YB, EC, OA, JK, YH, and SC: writing—original draft. All authors: writing—review and editing. All authors contributed to the article and approved the submitted version.

## Conflict of interest

The authors declare that the research was conducted in the absence of any commercial or financial relationships that could be construed as a potential conflict of interest.

## Publisher’s note

All claims expressed in this article are solely those of the authors and do not necessarily represent those of their affiliated organizations, or those of the publisher, the editors and the reviewers. Any product that may be evaluated in this article, or claim that may be made by its manufacturer, is not guaranteed or endorsed by the publisher.
