# Supplementary material for: Validation of the Grief Support in Healthcare Scale among frontline nursing professionals working in COVID-19 inpatient wards in Korea
Source: Front Psychiatry. 2023 Apr 20;14:1097022. doi: 10.3389/fpsyt.2023.1097022 (PMC10158932; doi:10.3389/fpsyt.2023.1097022)
Supplement: Supplementary file 1 [file Table_1.docx]

**Supplementary File 1**

**The Grief Support in Healthcare Scale (GSHCS)**

The following statements relate to the support that you receive. Please circle the response that best describes your level of agreement or disagreement with each statement.

SD – Strongly Disagree

D – Disagree

N – Neutral

A – Agree

SA – Strongly Agree

| Statement | Answer |
| --- | --- |
| 1. My family understands how close I am to the residents. | SD D N A SA |
| 2. My friends understand how close I am to the residents. | SD D N A SA |
| 3. My co-workers understand how close I am to the residents. | SD D N A SA |
| 4. My supervisors understand how close I am to the residents. | SD D N A SA |
| 5. Family members of the residents understand how close I am to the residents. | SD D N A SA |
| 6. My family knows that I have grief when residents die. | SD D N A SA |
| 7. My friends know that I have grief when residents die. | SD D N A SA |
| 8. My co-workers know that I have grief when residents die. | SD D N A SA |
| 9. My supervisors know that I have grief when residents die. | SD D N A SA |
| 10. Family members of the residents know that I have grief when residents die. | SD D N A SA |
| 11. My facility often holds memorial services for residents who have died. | SD D N A SA |
| 12. I am often able to attend memorial services inside my facility. | SD D N A SA |
| 13. I am often invited to attend memorial services outside of the facility. | SD D N A SA |
| 14. I am often able to attend memorial services for residents outside of the facility. | SD D N A SA |
| 15. My facility keeps me informed about the deaths of residents. | SD D N A SA |

Scoring: SD = 1; D = 2; N = 3; A = 4; SA = 5. Mean scores should be calculated. Higher scores indicate higher levels of grief support.

Note: The words “patient” and “patients” may be used in place of the words “resident” and “residents” when administering this scale in other health care settings.

**Supplementary File 2**

**의료인의 애도에 대한 지지 척도 (GSHCS)**

다음의 설명은 귀하가 받는 지지와 관련된 것입니다. 각 설명에서 귀하가 동의하거나 동의하지 않는 단계를 가장 잘 기술한 응답에 동그라미 하십시오.

SD – 전혀 동의하지 않는다

D – 동의하지 않는다

N – 보통이다

A – 동의한다

SA – 전적으로 동의한다

| 설명 | 답 |
| --- | --- |
| 1. 내 가족은 내가 거주인들과 얼마나 가까운지 이해하고 있다. | SD D N A SA |
| 1. 내 친구들은 내가 거주인들과 얼마나 가까운지 이해하고 있다. | SD D N A SA |
| 1. 내 동료들은 내가 거주인들과 얼마나 가까운지 이해하고 있다. | SD D N A SA |
| 1. 내 상관은 내가 거주인들과 얼마나 가까운지 이해하고 있다. | SD D N A SA |
| 1. 거주인들의 가족들은 내가 거주인들과 얼마나 가까운지 이해하고 있다. | SD D N A SA |
| 1. 내 가족은 거주인이 사망하면 내가 슬퍼한다는 것을 알고 있다. | SD D N A SA |
| 1. 내 친구들은 거주인이 사망하면 내가 슬퍼한다는 것을 알고 있다. | SD D N A SA |
| 1. 내 동료들은 거주인이 사망하면 내가 슬퍼한다는 것을 알고 있다. | SD D N A SA |
| 1. 내 상관은 거주인이 사망하면 내가 슬퍼한다는 것을 알고 있다. | SD D N A SA |
| 1. 거주인의 가족들은 거주인이 사망하면 내가 슬퍼한다는 것을 알고 있다. | SD D N A SA |
| 1. 내가 속한 시설은 사망한 거주인을 위한 추도식을 자주 연다. | SD D N A SA |
| 1. 나는 내가 속한 시설 내의 추도식에 자주 참석할 수 있다. | SD D N A SA |
| 1. 나는 시설 외부의 추도식에 참석하도록 자주 초대받는다. | SD D N A SA |
| 1. 나는 시설 외부의 거주인을 위한 추도식에 자주 참석할 수 있다. | SD D N A SA |
| 1. 내가 속한 시설은 거주인의 죽음에 대한 소식을 나에게 계속 알려준다. | SD D N A SA |

점수: SD = 1; D = 2; N = 3; A = 4; SA = 5. 평균 점수를 계산해야 합니다. 점수가 높을수록 슬픔 지원의 단계가 높다는 것을 나타냅니다.

참고: 다른 보건의료 환경에서 본 척도의 적용 시, “거주인” 및 “거주인들”이란 단어를 대신해서 “환자” 및 “환자들”을 사용할 수 있습니다.

**Supplementary Table 1. Measurement invariance across viral anxiety, depression, and generalized anxiety**

| **Model** | **χ^2^** | **Df** | **Δ χ^2^** | **Δdf** | **p** | **CFI** | **ΔCFI** | **RMSEA** | **ΔRMSEA** |
| --- | --- | --- | --- | --- | --- | --- | --- | --- | --- |
| **Having viral anxiety (SAVE-9 < 22 vs. SAVE-9 ≥ 22)** | | | | | | | | | |
| **Configural** | 45.104 | 68 |  |  |  | 1.000 |  | .000 |  |
| **Metric** | 48.164 | 76 | 3.060 | 8 | .931 | 1.000 | .000 | .000 | .000 |
| **Scalar** | 52.183 | 84 | 4.019 | 8 | .855 | 1.000 | .000 | .000 | .000 |
| **Having depression (PHQ-9 < 10 vs. PHQ-9 ≥ 10)** | | | | | | | | | |
| **Configural** | 39.199 | 68 |  |  |  | 1.000 |  | .000 |  |
| **Metric** | 50.542 | 76 | 11.343 | 8 | .183 | 1.000 | .000 | .000 | .000 |
| **Scalar** | 51.955 | 84 | 1.413 | 8 | .994 | 1.000 | .000 | .000 | .000 |
| **Having anxiety (GAD-7 < 10 vs. GAD-7 ≥ 10)** | | | | | | | | | |
| **Configural** | 45.312 | 68 |  |  |  | 1.000 |  | .000 |  |
| **Metric** | 52.637 | 76 | 7.325 | 8 | .502 | 1.000 | .000 | .000 | .000 |
| **Scalar** | 56.714 | 84 | 4.077 | 8 | .850 | 1.000 | .000 | .000 | .000 |

**Supplementary Table 2. Item fits and difficulties of the SAVE-9 scale through Rasch model**

| **Items** | | **Infit MnSq** | **Outfit MnSq** | **Difficulty** |
| --- | --- | --- | --- | --- |
| **Subscale 1 -**  **Recognition of the relationship** | **Item 1** | 1.02 | .99 | .31 |
|  | **Item 2** | .69 | .66 | .21 |
|  | **Item 3** | .95 | .91 | -.86 |
|  | **Item 4** | .88 | .82 | -.40 |
|  | **Item 5** | 1.46 | 1.49 | .74 |
| **Subscale 2 -**  **Acknowledgement of the loss** | **Item 6** | 1.04 | .99 | -.21 |
|  | **Item 7** | .71 | .65 | -.21 |
|  | **Item 8** | .93 | .87 | -.56 |
|  | **Item 9** | .84 | .75 | .06 |
|  | **Item 10** | 1.49 | 1.55 | .92 |
